# Integrated health reporting within the UN architecture: learning from maternal, newborn and child health

**DOI:** 10.1186/s41256-023-00342-x

**Published:** 2024-01-02

**Authors:** Svetlana Akselrod, Téa Collins, Daria Berlina, Amy Collins, Luke Allen

**Affiliations:** grid.3575.4 0000000121633745 WHO, Geneva, Switzerland

**Keywords:** Global health, United Nations, Health policy, Health governance, Maternal and child health, Sustainable development goals

## Abstract

Despite a proliferation of the United Nations General Assembly high-level meetings on a range of health issues and developmental challenges, global funding continues to flow disproportionately to HIV and maternal, newborn and child health (MNCH). Using the experience of MNCH, this short article argues that successful human rights framing and the development of robust and regular reporting mechanisms in the international development architecture has contributed to these areas receiving attention. Taking non-communicable diseases (NCDs) as an example of a relatively neglected health area, we propose mechanisms that would improve integrated reporting of health issues in a way that aligns with the move toward cross-cutting themes and matching political and financial commitments with impact. As new frameworks are being developed to support multi-agency approaches to achieving SDG 3—including reporting and accountability—there are opportunities to ensure MNCH and NCDs jointly seek data collection measures that can support specific targets and indicators that link NCDs with early childhood development.

## Introduction

Balancing the economic, social, and environmental dimensions of development, the 17 UN Sustainable Development Goals (SDGs) were launched in 2015 with 173 countries committing to the 2030 timeline. While health underpins each of the SDGs, Goal 3, “ensure healthy lives and promote well-being for all at all ages” laid out a set of specific targets that needed to be reached to achieve progress in health. A series of the United Nations General Assembly (UNGA) high-level meetings on health issues, including non-communicable diseases (NCDs), antimicrobial resistance (AMR), tuberculosis (TB), and universal health coverage (UHC), permeated the last decade of UNGAs, with a focus on garnering political and financial commitments, alongside linking health outcomes to the SDGs. Yet, the outcomes of these UNGA HLMs were mixed. Political declarations produced frameworks of commitment, but domestic prioritization of national plans, development assistance, national budgets, and programmatic outcomes have not universally materialized, and have not aligned with the burden of disease [1].

This paper considers how maternal, newborn and child health (MNCH) advocates have successfully used integrated reporting systems within the international development architecture to attract high levels of funding and political commitment. MNCH in this paper refers to a grouping of health conditions that affect mothers, newborns and children (e.g., neonatal preterm birth and maternal haemorrhage). Traditionally a major driver of disability and premature mortality in low- and middle-income countries, maternal and child health were focal to the Millennium Development Goals and continue to attract high levels of funding and political attention. The same is not true of many other areas, including NCDs. Why is this important? Because NCDs account for more than two thirds of all global deaths, and are a major driver of impoverishment and inequality [2]. It is vital that high-level commitments made at the UN actually translate into action within countries via funding and service delivery reform.

The ‘international development architecture’ is the system of institutions, policies and processes that are involved in promoting social and economic development around the world. This architecture is comprised of United Nations (UN) agencies, multilateral development banks, bilateral aid agencies, international non-governmental organisations and a range of other actors including research institutions and philanthropic foundations. The architecture also includes frameworks and agreements around trade, health regulations, initiatives such as the SDGs, and reporting and accountability structures. In this article we argue that systematic and integrated reporting systems can help to attract funding and political prioritization for noncommunicable diseases (NCDs) and other important health issues that have failed to attract adequate funding and/or political attention [3], using the MNCH advocacy model.

## Learning from the success of MNCH and HIV

Development Assistance for Health (DAH) funding—i.e. money from international donors—continues to be channelled largely to MNCH and HIV in low- and middle-income countries where strong multidimensional approaches to accountability exist. In both cases, political commitment has translated into political and financial commitments and positive health and development outcomes across the global south [4, 5]. For HIV, this takes the form of a UN Declaration of Commitment and annual review of HIV progress at the UN General Assembly. For MNCH, a three-pronged ecosystem has been developed that comprises financing (through the Global Financing Facility), technical support (through the ‘H6’ partnership of six different UN institutions) [6], and accountability and alignment (through the Partnership for MNCH). All three elements underpin the landmark ‘Every Woman Every Child’ initiative [7]).

One of the unique elements central to the success of MNCH advocates is the centrality of human rights within their global strategies and the requirement for accountability based on human rights approaches. For both HIV/AIDS and maternal, newborn and child health, regular integrated monitoring and reporting have been another central facet of holding governments accountable to their commitments.

In contrast, NCDs have not been framed as an issue of human rights. Inequalities are often mentioned, however the argumentation is frequently flawed: for instance, the power of the oft-repeated fact that 74% of premature NCD deaths occur in low and middle income countries is undermined by the fact that 80% of the world’s population live in these countries [8]. Whilst donor funding is often channeled to discrete, solvable issues with widely available data, NCDs are a heterogenous collection of complex problems that require multisectoral action [3]. Until relatively recently, NCDs were conceived as self-imposed diseases of the rich that require individual-level solutions, rather than socially transmitted conditions that disproportionately burden the disadvantaged [9]. Whilst the MNCH global strategy has institutionalised support from six UN agencies, it’s own financing facility, and the UN Secretary-General as it’s booster, the WHO NCD Global Strategies have none of the above, nor routine reporting commitments to the UNGA.

The UNGA High-Level Meetings provide an opportunity to raise the profile of NCDs. These meetings started to proliferate in the 2000s after the first UNGA special session on HIV/AIDS, and each one brings a new set of political commitments. There have been 16 special sessions and High-Level Meetings on health topics since 2000, including three in September 2023. The commitments for NCDs, AMR, UHC and TB all place them within the UN Global Health and Foreign Policy agenda, which involves an annual meeting of member state representatives. However, reporting back to the UNGA only happens for these conditions when called for in disease-specific declarations. While UHC and NCDs both have High-Level meetings in 2023 and 2025 respectively, the lack of annual reporting to the highest level of the UN system (i.e. UNGA) makes it difficult to sustain strong political commitment, accountability and funding. As progress on the SDGs is reported every year through the UN high-level political forum (UNHLP), there are opportunities within the current architecture to integrate the reporting of MNCH and NCDs.

## Common underlying factors between NCDs and MNCH

NCDs and MNCH overlap in a number of areas. Conditions in the womb can expose foetuses to a range of health challenges and puts them at increased odds of developing hypertension, diabetes, chronic renal impairment, and heart disease, along with mental health conditions, due both to predisposition and intergenerational transmission [10, 11]. Births of premature children, not fully grown, and to a mother that is overweight or has diabetes represent over 50% of all births globally [10]. The interventions to address both conditions remain largely at the primary health care level, which can act as the point for education, awareness, screening and treatment and has been identified as central to achieving UHC. The framing of NCDs as conditions that can be acquired has enabled dialogue and support through social protection measures, including interventions for key populations such as women and children. Social protection measures are often mandated in national constitutions [12] and provide a more holistic and coordinated approach to addressing social, economic, and environmental factors that facilitate poor NCD and MNCH outcomes. The ‘life course approach’ is a helpful unifying framework for joint action on MNCH and NCDs. It stresses the overlapping stages, transitions, and settings where large improvements can be made to promote health, recognising that all stages of life are intertwined [13].

The UNGA high-level meetings established that there are similar underlying challenges plaguing each health sector area and that mechanisms to monitor and report back are critical to ensuring national prioritization. It has also shown that investment in human capital, resilience building and one-health approaches need to underpin disease-specific plans. The launch of the Global Action Plan for Healthy Lives and Well-being for All signalled this shift with 13 agencies coming together to align ways of working and provide a more streamlined approach to health and development frameworks and programme implementation. Where do the NCD and MNCH agendas sit in the health and development architecture and how can a new more coordinated health ecosystem support accountability on investment and policy implementation?

Integration, linkages and synergies among different health programmes, whether HIV/AIDS, TB, MNCH, sexual and reproductive health, UHC, or NCDs, have long been advocated for [14, 15]. As a more recent health focus area with less developed health systems infrastructure in place and diverse interventions, NCDs have been identified as an area that can balance horizontal and vertical planning, budgeting and health service implementation to achieve synergistic benefits [14].

### Context and framing

With positive progress on women's and children’s health closely linked to gender equality and humanitarian recovery, the ‘Every Woman Every Child’ movement launched by the UN Secretary-General himself, and the UN MNCH Global Strategies emphasise various levels of accountability; contexts, settings, risks and crises; and the need for conceptual policy and programme opportunities that bring actors from all sectors [16]. It galvanized political leadership with 776 commitments recorded, totalling over US$186 billion, and saw domestic spending on MNCH increase, with a quarter of financial commitment makers coming from low-income countries, the private sector and non-governmental organizations [17–19]. The building blocks for this achievement have been strong monitoring and tracking, along with accountability components founded in a human rights approach [20]. The 60 indicators, all commitments—financial, policy, as well as the service and delivery—are aligned, tracked, and reported on, leading to concrete policies, plans and strategies with budgets [21]. Tracking existing country commitments to health and human rights, along with opportunities for existing or enabling legislation, have also been monitored to ensure the continuity of commitment. This approach has also been seen as necessary to empower and promote well-being [22, 23].

Accelerating health commitments, specifically those set out in SDG 3 will be supported through the implementation of the Global Action Plan on Health and Well-being for All. This identifies seven areas of focus: primary health care, sustainable finance, fragile settings, determinants of health, community and civic engagement, gender equality, research and development, and data and digital—which has translated into implementation in 37 countries [24]. Focusing on selected areas of action, provides an opportunity for synergy, ensuring primary health care essential services include MNCH and NCD interventions, strengthening country data that can capture needed areas for the policy or programmatic implementation, and scaling up innovations [25].

Yet, there has also been a call by the Independent Accountability Panel of the Every Woman Every Child movement for the institutionalization of an Independent Review Mechanism that reports on health across the SDGs. As the mandate of the Independent Accountability Panel ended in 2020 after 5 years of annual reporting on progress and monitoring commitments to hold stakeholders accountable, the need to reframe health as a central feature of achieving the SDGs and linking it to a framework of rights was called for. The independent review mechanism would report and input into the high-level political forum (HLPF) on sustainable development. The HLPF meets annually at the UN headquarters in New York, with member states, representatives from civil society, the private sector, and other stakeholders coming together to review progress on the SDGs. The forum takes place over 8 days, three of which are ministerial level. Once every 4 years the HLPF is held in the plenary of the UN General Assembly at the level of the Head of State and government.

The HLPF encourages country reporting through voluntary national reviews. An accountability mechanism—as laid out by the Independent Accountability Panel—can support accountability in several ways: It can look at legal foundations; it can support the design and structures of pathways that are critical for operationalizing accountability (by enabling political, economic and sociocultural adjustments); it can promote organizational processes that better serve citizens such as health assemblies [26], health facility charters [27], and channels that citizens can participate in such as community scorecards; as well as social audits and budgeting [28].

### Reporting through the high-level political forum

There are an increasing number of countries that provide information on their progress towards the SDGs within the High-Level Political Forum, with 126 countries or territories reporting since 2016 [29]. For countries, the reporting process is often linked to the UN Sustainable Development Framework, which is the central framework for joint monitoring, review, reporting and evaluation of the UN development system’s impact on a country towards achieving the 2030 Agenda [30]. Of the 134 UN Country Team offices, 53 of them have cross-agency frameworks according to the website, with more in development [31, 32]. Of the 53 with frameworks, 51 provided voluntary reports during an annual HLPF. As such, these reporting mechanisms will continue to strengthen, with the opportunity to align to WHO country cooperation strategies and the Global Action Plan for Health and Wellbeing—all of which will set overarching priorities for countries—and feed into strategies and budgeting for long-term national development and sustainability, such as Sri Lanka’s experience (Box 1).

**Fig. 1:**
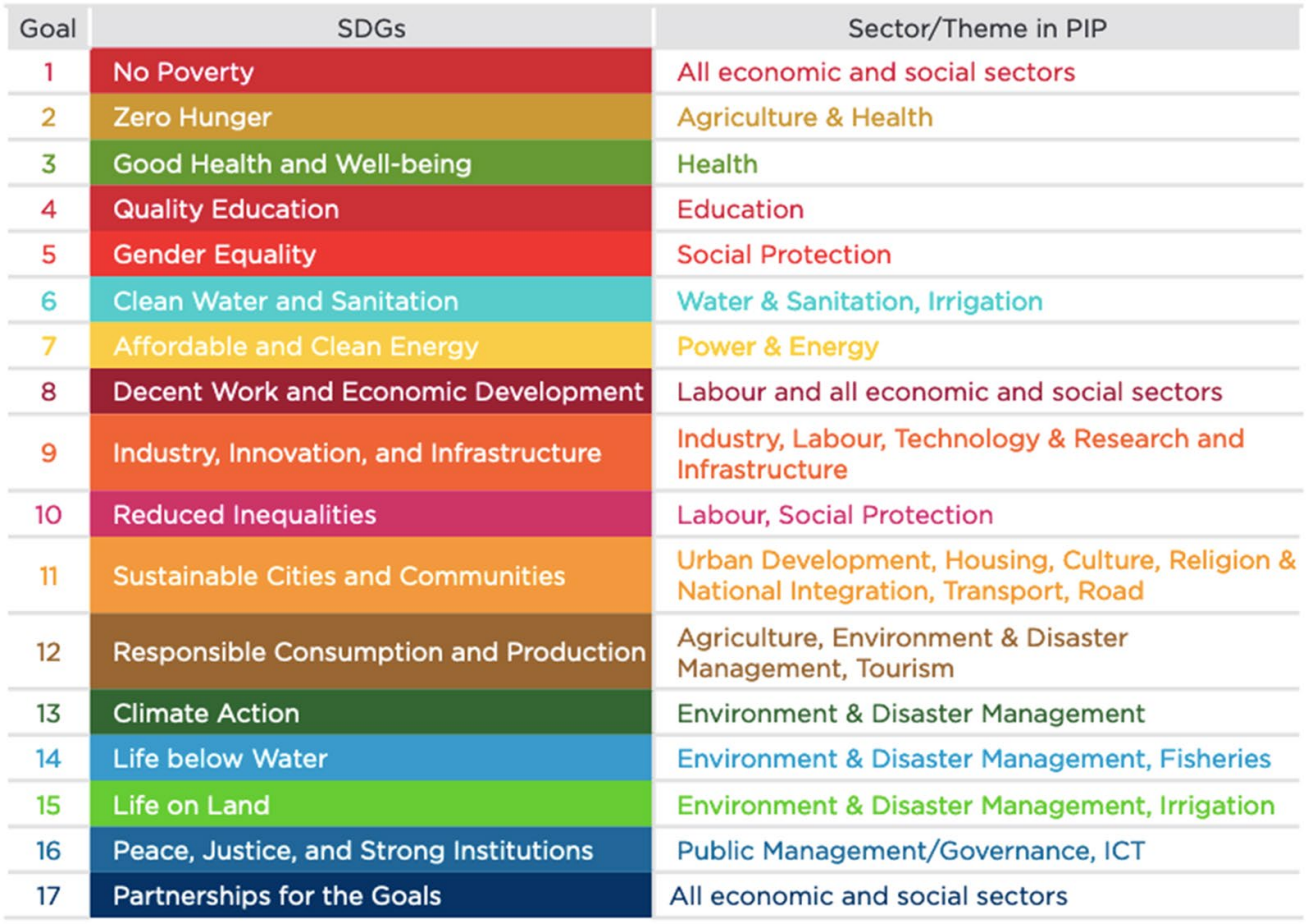
Sri Lanka’s SDG mapping for public investment

Box 1: Sri Lanka’s experience Sri Lanka has aligned its 2018–2022 WHO Country Cooperation Strategy [33] to the Sustainable Development Goal Framework [34], establishing its national priorities and aligning funding with the SDGs. This has been done through the establishment of a Sustainable Development Council with efforts to improve data availability on the SDG indicators. The integrative approach indicates their strong leadership in the area, however, in its report to the HLPF in 2018, it maps the key areas of public investment, with health directly mentioned only under SDG 2 and SDG 3 (Fig. 1) [35]. As each of the goals requires a strong and resilient health and social system, using the existing linkages of the MNCH movement within other SDGs to NCDs, and ensuring these are firmly anchored within economic and social plans through a human rights lens could be further developed.

## Opportunities for integrated reporting for NCDs and MNCH

As the prioritization of thematic health areas is being accelerated under the *Global Action Plan for Healthy Lives and Well-Being for All* for SDG 3, there are opportunities for MNCH and NCDs to integrate monitoring and reporting using existing data sources and collection methods under other SDG goals and aligning with specific targets and indicators. This can offer opportunities to strengthen plans that require political, financial, and programmatic commitments to health, and health and social determinants.

To use an example from TB reporting, data have been collected on the number of cases attributed to select risk factors, including alcohol use disorders, diabetes, HIV, smoking and undernourishment, giving a baseline to monitor achievement when linking with other disease programme areas. It has also linked achievement of TB outcomes to areas outside of SDG 3, looking at decreasing TB incidence by reducing catastrophic costs through social protection measures (SDG 1.3.1) addressing undernourishment (SDG 2.1.1), and looking at the pathways within and outside of their TB health metrics systems. Prior to the UN High-Level Meeting on TB, an SDG-TB framework was developed that identified existing reporting streams and metrics that can be monitored to inform progress towards targets set out in the ‘End TB Strategy’ [36, 37]. This framework roots the success of TB outcomes to larger social, economic, and political forces measured in the SDG targets. Similarly, AMR has convened a Global Leaders Group which will build on existing political commitments from the G7 and G8, and World Bank, to build conducive and enabling policy environments [38].

While there are numerous targets and indicators linked to MNCH, there is an opportunity to further develop the NCD indicators that look at preventative measures that can be achieved in partnership with MNCH to support NCD outcomes along the life course. These include addressing HPV through vaccination (and using vaccination as an opportunity to educate through multipurpose campaigns); awareness campaigns on nutrition for obesity and alcohol; looking at the built environment to enable communities that support physical and mental health and governance; amongst others. Demand for NCD and MNCH integration continues to increase as monitoring and reporting on the quality of care and effective coverage (NCDs are one of the tracer indicators for UHC), nutrition programmes, early childhood development and conflict settings become more central spaces for interventions [39].

## Conclusions

In comparison to MNCH, global efforts to tackle NCDs have been underfunded, misdirected, and underwhelming. The successes in addressing MNCH mortality over the last three decades are partly attributable to the establishment of regular reporting mechanisms to the highest levels of the UN and the integration of health reporting for these conditions alongside other health, social, and economic indicators. There are upcoming opportunities to use these lessons to increase funding and political attention for more overlooked areas such as NCDs whilst stressing the synergies that can be gained from addressing shared risk factors. These include advocating for shared targets and indicators within the SDG 3 framework and the use of existing UN frameworks to support the inclusion of reporting and accountability on integrated NCD and MNCH approaches for voluntary national review processes. Examples include the WHO Collaborating Centres Strategy and the UN Sustainable Development Frameworks. Identifying key policy interventions that are country-specific, and supporting the reporting of these interventions can allow for the development of a more comprehensive MNCH and NCD infrastructure that aligns targets and indicators and sets out a roadmap for enabling environments that take a life course approach. In terms of the broader international development architecture, NCDs would benefit from the elevation of current reporting to the UNGA main session; greater UN inter-agency collaboration on the development and implementation of the next Global Action Plan, ideally launched by the Secretary-General; and from the creation of a dedicated financing facility to catalyse and align funding for these overlooked yet critically important conditions.

## Data Availability

All data generated or analysed during this study are included in this published article.

## Abbreviations

AMR: Antimicrobial resistance
HLM: High-level meeting
HLPF: High-level political forum
MNCH: Maternal, newborn and child health
NCDs: Non-communicable diseases
SDGs: Sustainable development goals
TB: Tuberculosis
UNGA: UN General Assembly
UHC: Universal health coverage
